# Pyosalpinx as a sequela of labial fusion in a post-menopausal woman: a case report

**DOI:** 10.1186/1752-1947-5-546

**Published:** 2011-11-06

**Authors:** George I Tsianos, Stefania I Papatheodorou, George M Michos, George Koliopoulos, Theodor Stefos

**Affiliations:** 1 Department of Obstetrics and Gynecology, University of Ioannina School of Medicine, Ioannina 45110, Greece

## Abstract

**Introduction:**

Complete labia fusion is a rare clinical entity in post-menopausal women. The most common complications of this presentation are infections of the urinary tract and retention of urine in the vagina. We present the case of a post-menopausal woman with adnexal mass and abdominal pain due to fusion of the labia majora. To the best of our knowledge this is the first report in the literature of this complication.

**Case presentation:**

A 78-year-old Caucasian woman was admitted to our hospital due to abdominal pain and urination difficulty, along with fever and leucocytosis. On examination the labial majora were fused. Computed tomography of the abdomen revealed a cystic formation in the anatomical area of the right adnexa. Our patient had developed a pyosalpinx as a Sequela of labial fusion. At laparoscopy the right pyosalpinx was identified and resected, whereas the labia majora were reconstructed via dissection and separation.

**Conclusions:**

Labial fusion is a rare clinical entity in post-menopausal women and can have serious and unexpected complications. Though this presentation is rare, a clinical examination must be performed in detail in order to gain valuable information for an accurate diagnosis. Post-operational instruction must be given to patients in order to prevent the re-occurrence of the fusion and its complications.

## Introduction

Labial fusion in adults is a rare clinical entity with only few described cases, in particular in post-menopausal women. The major symptom in those reported cases is urination anomalies and most commonly infections of the urinary tract. Pyosalpinx is most often a complication of pelvic inflammatory disease arising from a variety of causes. In post-menopausal women, pyosalpinx is commonly associated with primary fallopian tube malignancy. We report a case of a post-menopausal woman who developed a pyosalpinx following complete fusion of the labial majora.

## Case presentation

A 78-year-old Caucasian Greek post-menopausal woman was admitted to our hospital due to continuous pain in her lower abdomen along with fever. Both symptoms began one week prior to admission and had become progressively worse over the 24 hours before presentation when she experienced four vomiting episodes. Our patient reported a long-standing history of urinary incontinence and voiding difficulty.

**Figure 1:**
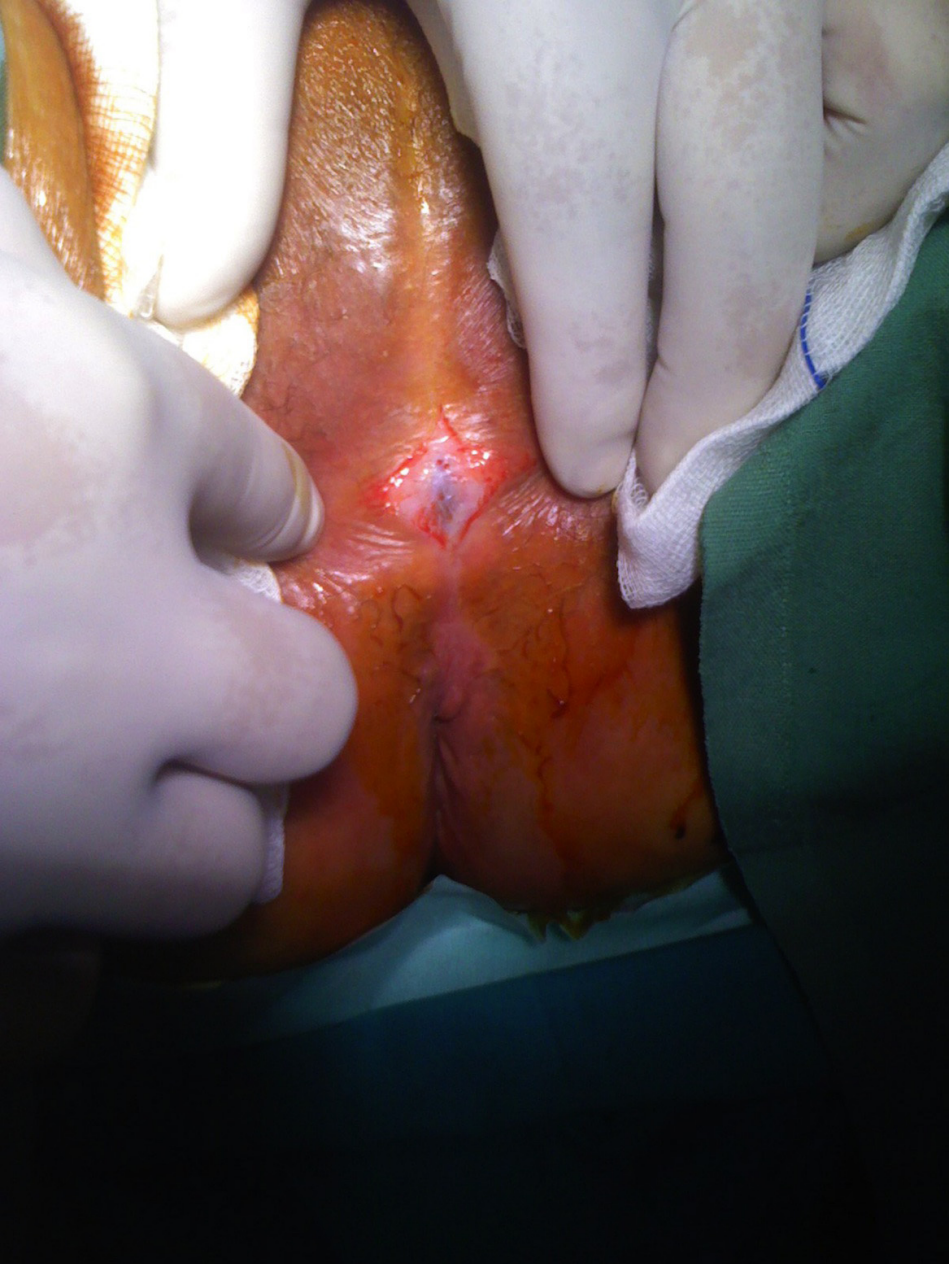
**Labial fusion: pre-operational image of labial fusion**. The labia majora are completely fused, leaving a very small opening to allow urination.

Her overall clinical examination did not reveal anything worrisome, although a gynecological examination was unfeasible due to complete labial majora fusion (Figure 1). There was no visible opening that would allow urination, although our patient mentioned being constantly wet. Our patient recalled a similar labial fusion in the past, which had been surgically treated six years previously; unfortunately neither the records nor the means of treatment were available from her previous hospital admission. Her medical history revealed coronary heart disease and type II diabetes mellitus. She had been a widow for over 20 years, and claimed to have had no sexual relationships since her husband's death.

Laboratory blood analysis on admission revealed the following: leucocytosis (white blood cell (WBC) count 13.02 × 109/L) with neutrophilia (neutrophils 11.95 × 109/L) with a further rise on day two (WBC: 18.64 × 109/L) followed by a drop on day four (WBC: 12.82 × 109/L); an elevated C-reactive protein level (105 mg/L) with a further rise on day two (119 mg/L) followed by a drop on day four (59 mg/L); an elevated blood glucose level (165 mg/dL) followed by a drop on day two (142 mg/L) and then the level remained within normal limits throughout the rest of her hospital stay and finally an elevated lactate dehydrogenase level (286 U/L). Carcinoembryonic antigen (CEA), α-fetoprotein (AFP), β-human chorionic gonadotropin (β-HCG), cancer antigen 125 (CA 125), cancer antigen 19.9 (CA 19.9) and cancer antigen 15.3 (CA 15.3) levels were all normal. Serum urea, creatinine, sodium and potassium levels were within normal limits. A computed tomography (CT) scan of her abdomen on admission showed a cystic (7 × 4.5 cm) formation within the pelvis minor in the anatomical position of the right adnexa; the formation appeared to be tubular in shape most likely suggesting a hydrosalpinx or pyosalpinx.

After assessing all available evidence we were led to believe that our patient had most likely developed a pyosalpinx as a complication of the labial majora fusion. Our patient was started on antibiotic treatment. Three days later she underwent a minor surgical procedure during which a small opening was made in the outer vulvar lips in order to assist urination. Following antibiotic treatment there was resolution of her symptoms and, as our patient was judged to be at high risk if placed under general anesthesia, a conservative treatment of the pyosalpinx was preferred. A week later our patient was discharged and an appointment for a follow-up visit was made in two weeks. During the scheduled appointment a decision would be made on whether to proceed to labial separation alone or combined abdominal surgical treatment of the pyosalpinx.

On arrival for the follow-up visit her blood test results were normal except for her blood glucose (174 mg/dL) and lactate dehydrogenase (257 U/L) levels. Her CEA, AFP, CA 125, CA 19.9 and CA 15.3 levels were normal; β-HCG however was mildly elevated. A second CT scan of the abdomen confirmed persistence of the previous pyosalpinx finding. Furthermore, a 7 cm elongated cystic formation suggestive of a cystovaginal fistula was identified, as well as bilateral mild hydronephrosis as a result of the urinary bladder's over-distension.

**Figure 2:**
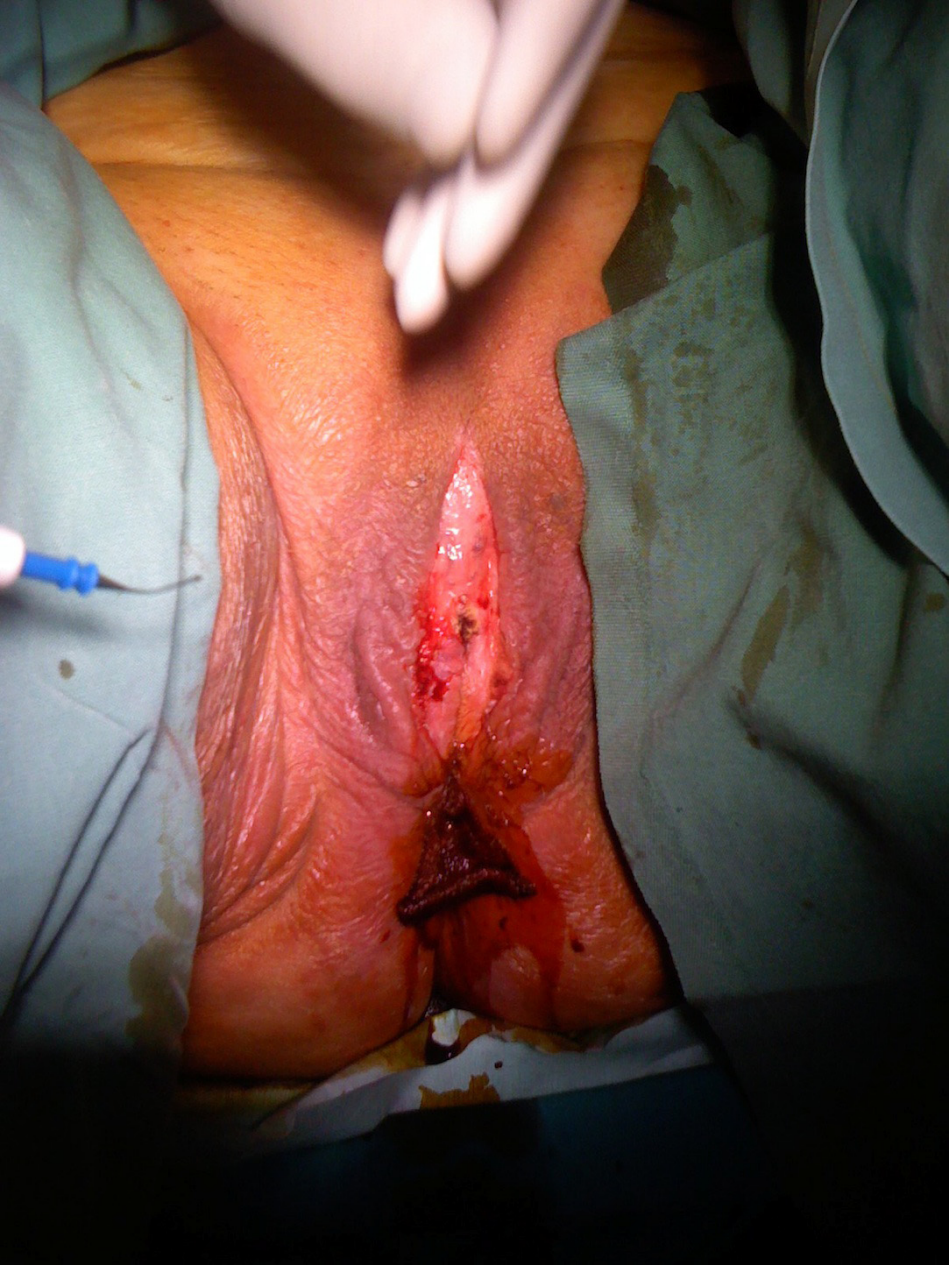
**Dissected labia majora**. Post-operative appearance of the labia after the blunt dissection and separation.

**Figure 3:**
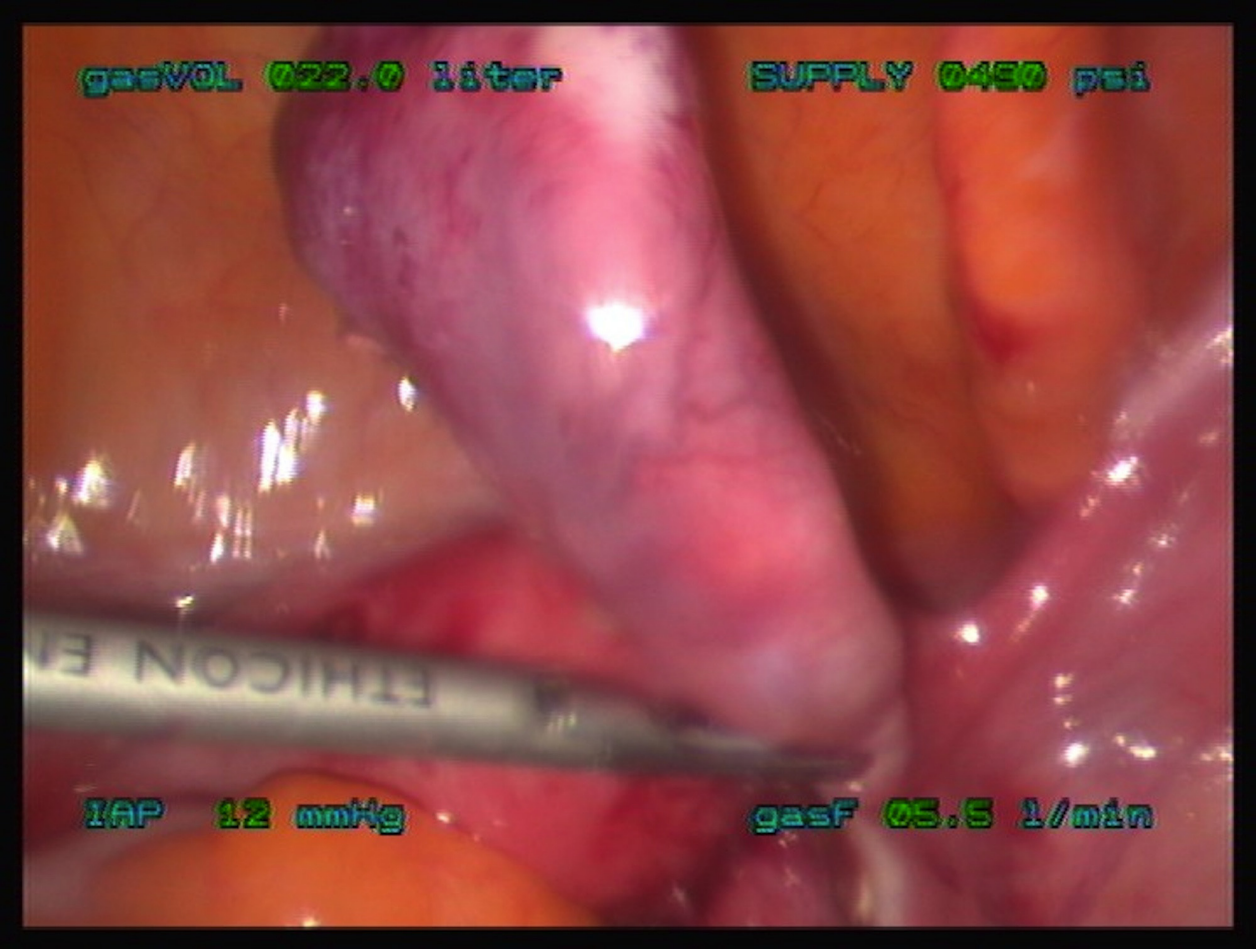
**Pyosalpinx**. Intra-operative image of the fallopian tube during laparoscopic resection. The pyosalpinx was removed intact.

On day two of admission our patient underwent laparoscopic surgery during which the right pyosalpinx was identified and subsequently resected (Figure 2), and in the end the labia majora were reconstructed via blunt labial dissection and separation (Figure 3). During the operation, we also identified accumulation of a large amount of urine in the vagina, suggestive of urocolpos, which was observed on the CT scan but no signs of a urinary fistula were seen. Our patient was discharged two days later without any problems or complications. Five months following the procedure our patient is on regular follow-up without any symptoms. She has been advised to regularly use vaginal dilators and courses of local estrogen treatment.

The pathology report from the tissue specimens retrieved from the operation showed acute and chronic inflammation in the fallopian tube mucosa and chronic inflammation in the skin of the labia majora. No signs of malignancy were found in either of the fallopian tubes or the labia majora.

## Discussion

Pyosalpinx following labial fusion has not been reported previously in the literature and, to the best of our knowledge, this is the first case report of such Sequela. Labial fusion occurs mainly in the first years of life and can be congenital or acquired. The condition is rare in adults and only a few cases have been reported in the literature, predominantly in post-menopausal women. The pathophysiological mechanism is believed to be chronic inflammation and/or irritation of the vulvar skin arising from various conditions such as local inflammation [1], lichen sclerosus [2], recurrent urinary tract infections [3] as well as lack of sexual activity, lack of estrogen and local trauma. The major presenting symptom is urinary incontinence [1,4,5] and voiding dysfunction [6,7] whereas the mechanism by which this occurs is most likely due to physical obstruction of the urinary flow, directly related to the labial fusion. In addition, the mechanical obstruction of urine flow commonly results in a retrograde flow inside the vaginal cavity, forming a urocolpos and thus ultimately leading to urination anomalies; such cases have been previously described [8,9].

Our patient had been previously diagnosed with labial fusion and although she recalled having been surgically treated, her condition reappeared. Recurrence of labial fusion has been previously described [10], and we assume that in our patient, the fused labia during the first episode may not have been significantly surgically separated. In addition, it seems that she was not given any post-operative instruction for use of vaginal dilators and local estrogen treatment.

These instructions in our view are essential for the maintenance of a good outcome. In our patient although there was no visible vulvar opening, it is likely that some amount of the urine that was accumulating inside the vagina causing urocolpos was constantly filtered through the fused labia, since our patient stated she was continuously wet.

A pyosalpinx is one of the features of pelvic inflammatory disease and refers to the presence of pus in one of the fallopian tubes occurring as a consequence of an infection in the reproductive tract. Infections may start in the vagina and progress up to the cervix, uterus and to one or both fallopian tubes. The most notable long-term complications of this condition are tubal infertility and ectopic pregnancy. The most common causative microorganisms are *Chlamydia trachomatis*, *Neisseria gonorrhea*, and microorganisms associated with bacterial vaginosis [11], although other causes have been described in the literature such as infections by *Streptococcus pneumoniae *[12], coliforms [13], or even complications during *in vitro *fertilization [14], and appendectomy [15].

In the present case report it is highly probable that due to the urine accumulation in the vaginal cavity the microorganisms in the formed urocolpos ascended through the cervix, inside the uterus and then up the fallopian tube where an infection formed and a pyosalpinx ultimately developed. Pyosalpinx can be treated conservatively with antibiotic treatment but surgical intervention in the form of salpingectomy might be required; in the present case report, this was performed laparoscopically.

## Conclusions

It is clear that complete or partial labial fusion can result in urinary tract obstruction and infection to varying degrees. Clinical identities that are not common in a specific age or risk group should always be included in a differential diagnosis in order to obtain a complete and effective therapeutic approach for each patient. In this particular case, a pyosalpinx was formed not due to a pelvic infection as may be expected in a younger and sexually active patient, but due to mechanical obstruction of the outflow of urine. Combining all data from clinical examination and imaging techniques can lead the physician to apply an evidence-based therapeutic strategy.

## Patient's perspective

[Translated from the patient's native language] In one of the follow-up visits, I was asked about my feelings regarding my problem. I mentioned that I found not being able to urinate properly distressing and I was embarrassed to be constantly wet. I was disappointed that the initial procedure some years ago failed and that I had not been instructed to be under regular follow-up, nor had I been given instructions to try and keep the vulvar introitus open. I was extremely pleased with the outcome of the second procedure. I am happy that I am able to completely empty my bladder and was surprised I recovered so quickly from the laparoscopic salpingectomy. I am very willing to do whatever I am instructed to prevent a recurrence of the fusion, and I agree to have my case shared with the medical community if it helps other women with a similar problem.

## Consent

Written informed consent was obtained from the patient for publication of this case report and any accompanying images. A copy of the written consent is available for review by the Editor-in-Chief of this journal.

## Competing interests

The authors declare that they have no competing interests.

## Authors' contributions

GIT, SIP and GMM analyzed and interpreted the data from our patient regarding clinical presentation, and hematological and imaging data. GK and TS performed the first labial dissection and the operative laparoscopy. All authors contributed to writing the manuscript and approved the final version.
